# NADPH oxidases: Pathophysiology and therapeutic potential in age-associated pulmonary fibrosis

**DOI:** 10.1016/j.redox.2020.101541

**Published:** 2020-04-17

**Authors:** Kosuke Kato, Louise Hecker

**Affiliations:** a Department of Medicine, Division of Pulmonary, Allergy and Critical Care and Sleep Medicine, University of Arizona, Tucson, AZ, 85724, United States; b Southern Arizona VA Health Care System (SAVAHCS), Tucson, AZ, 85723, United States

## Abstract

Oxidative stress has been associated with a number of human fibrotic diseases, including idiopathic pulmonary fibrosis (IPF). Although oxidative stress is associated with both fibrosis and aging, the precise cellular sources(s) of reactive oxygen species (ROS) that contribute to the disease pathogenesis remain poorly understood. NADPH oxidase (Nox) enzymes are an evolutionarily conserved family, where their only known function is the production of ROS. A growing body of evidence supports a link between excessive Nox-derived ROS and numerous chronic diseases (including fibrotic disease), which is most prevalent among the elderly population. In this review, we examine the evidence for Nox isoforms in the pathogenesis of IPF, and the potential to target this enzyme family for the treatment of IPF and related fibrotic disorders. A better understanding of the Nox-mediated redox imbalance in aging may be critical to the development of more effective therapeutic strategies for age-associated fibrotic disorders. Strategies aimed at specifically blocking the source(s) of ROS through Nox inhibition may prove to be more effective as anti-fibrotic therapies, as compared to antioxidant approaches. This review also discusses the potential of Nox-targeting therapeutics currently in development.

## 1 Introduction

The ultimate function of the lungs is to facilitate the diffusion of gases. This occurs primarily through the exchange of carbon dioxide (CO_2_) for oxygen (O_2_) across alveolar-capillary membranes. Adult human lungs exchange 10,000–20,000 L of air daily [1]. Oxygen is essential for complex biological life, as it is fundamental to cellular metabolism and energy production. The breakdown of oxygen is a by-product of aerobic metabolism, which leads to the production of reactive oxygen species (ROS), including superoxide radical anion (O_2_^•−^) and hydrogen peroxide (H_2_O_2_). ROS are produced by various cell types in the lung, including myofibroblasts, epithelial, inflammatory, endothelial, and smooth muscle cells. These highly reactive intermediates play pivotal roles as signaling molecules to regulate a wide variety of physiologic functions. For example, ROS signaling is involved in numerous cellular processes, including DNA stability, cellular senescence, apoptosis, and extracellular matrix (ECM) remodeling [2,3]. All of these ROS-mediated cellular processes are important during normal repair responses.

Despite the critical physiological roles of ROS, redox imbalance leading to oxidative stress is associated with deleterious biological consequences. Oxidative stress occurs when cellular ROS levels overwhelm antioxidant capacity. Such a state of redox imbalance may lead to the damage of cellular macromolecules (e.g., DNA, lipids, and proteins) and/or cytokine production, which may ultimately result in organ dysfunction. Several enzymes in the body are capable of producing cellular ROS, including lipoxygenases, nitric oxide synthase, xanthine oxidase, cytochrome P450 oxidases, mitochondrial electron transport chain, and NADPH oxidases (Nox) [4]. However, a majority of these enzymes produce ROS as a by-product of their catalytic activities. In contrast, Nox produce ROS as their primary and sole function. Since oxidants can act indiscriminately to modify biomolecules, cellular antioxidant systems have evolved to maintain redox balance. These cellular antioxidant sources include superoxide dismutases, catalases, peroxiredoxins, and glutathione systems [5,6]. A growing body of evidence supports a link between excessive Nox-derived ROS and numerous chronic diseases (including fibrotic disease) [4], which tends to appear late in life. The current review is focused exclusively on Nox, which are a major cellular source of ROS generation, and their roles in the pathogenesis of pulmonary fibrosis.

Fibrosis in mammalian tissues is an evolutionarily conserved and adaptive response to injury. Physiological fibrosis is a complex biological trait that permitted survival of vertebrates as they transitioned from aquatic to terrestrial habitats and from cold-blooded to warm-blooded physiology. Survival of these early vertebrates likely necessitated the capacity for a wound healing-fibrosis response. For example, fibrosis can be protective as an effective barrier to prevent rapid blood loss or to prevent the spread of pathogens. Similar “beneficial” roles for fibrosis may be argued for such fundamental processes as the plant hypersensitivity response (by crosslinking of proteins to form a “scar” around pathogens and limit their invasion) [7,8]. This evolutionarily conserved mechanism likely explains the “walling off” of bacterial pathogens (lung abscesses) and host defense responses to limit the spread of *Mycobacterium tuberculosis* and fungal infections (commonly associated with fibrosis surrounding these organisms) [8]. Thus, fibrosis could be viewed as an “evolutionary trade-off” that allowed for the survival of the species against infectious agents, albeit at the expense of loss of tissue structure/function [8]. However, the enigma is understanding how/why this evolutionarily conserved process goes awry, leading to the pathogenesis of fibrotic disease. Numerous studies have demonstrated critical roles for Nox in mediating physiological fibrotic responses to injury (reviewed below). However, excessive Nox-dependent ROS has also been implicated in a variety of fibrotic diseases, including the skin [9], liver [10], pancreas [11], kidney [12], heart [13,14], and lung [[15], [16], [17]]. It has been speculated that Nox represents “antagonistically pleiotropic” genes – they confer a reproductive advantage in early life, but have harmful effects late in life [8,18].

### 1.1 IPF pathophysiology and epidemiology

Idiopathic pulmonary fibrosis (IPF) is a relentlessly progressive and fatal disorder affecting 130,000 people in the United States and ~3 million worldwide [19]. IPF is characterized by excessive scar tissue formation and destruction of lung parenchyma, resulting in gas exchange abnormalities and ultimately respiratory failure. The median survival rate for IPF patients is less than 3 years [20,21]. The key pathological hallmarks of IPF are the accumulation of myofibroblast clusters (fibroblastic foci) and a loss of alveolar epithelial cells.

By definition, IPF has an unknown etiology (i.e., idiopathic), although a number of risk factors have been identified. Studies support the role of intrinsic risk factors (genetics, age, sex, microbiome), co-morbidities (gastroesophageal reflux, obstructive sleep apnea, diabetes mellitus, viral infection), and extrinsic risk factors (cigarette smoke, environmental exposures, air pollution) in IPF development [22]. However, the role of aging stands out as a major contributor to IPF pathogenesis, and IPF is widely regarded as a disease of aging [[23], [24], [25]]. Numerous studies have provided evidence to support the concept that IPF represents an age-related degenerative disease process [26]. Both familial and sporadic cases of IPF have been associated with telomere shortening [27,28]. The cause(s) for the shortened telomeres in IPF patients without mutations in telomerase is currently unknown; however, oxidative stress represents one potential mechanism. Several other hallmarks of aging have also been reported in IPF lungs. In general, progressive fibrosis is a hallmark of aging in various organ systems, including the liver [29], kidney [30], pancreas [31], and lung [32]. The incidence and prevalence of IPF increase with age. Two-thirds of IPF patients are >60 years of age when the disease initially presents, with a mean age of 66 years at the time of diagnosis [21]. Further, the survival rate of IPF patients markedly decreases with advancing age [24]. With a growing elderly population, it has become increasingly important to understand the age-associated mechanisms that contribute to disease pathogenesis.

### 1.2 Age-associated redox imbalance in IPF

Aging and fibrotic disease are both associated with cumulative oxidant burden, and oxidative stress has been implicated as a key mediator in the pathogenesis of IPF. IPF patients exhibit significantly elevated oxidative stress biomarkers in exhaled breath condensate, including H_2_O_2_ and 8‐isoprostane [33]. Further, bronchoalveolar lavage fluid (BALF) from IPF patients exhibit elevated levels of oxidative damage to proteins, characterized by the introduction of carbonyl groups into amino acid side-chains [34] and oxidation of methionine to methionine sulfoxide by free radicals [35]. Lung tissue from IPF patients also demonstrate signatures of chronic oxidative damage [36,37]. Oxidative changes within the lung may provide a positive-feedback mechanism for perpetuating a pro-fibrotic tissue microenvironment by mediating various cellular behaviors that influence repair responses [38]. Oxidative stress may represent a core pathway by which damage theories of aging are based. Examples include genomic instability or senescence as a result of oxidative stress-associated DNA damage. Senescence is broadly defined by irreversible cell-cycle arrest accompanied by profound phenotypic alterations, including a senescence-associated secretory phenotype (SASP), a key feature of which is elevated levels of secreted ROS [39]. However, redox imbalance in itself, due to excessive ROS or antioxidant deficiencies, can also lead to the induction of senescence. The incidence of cell senescence increases with advancing age, as senescence is induced by various pro-aging stressors, including telomere attrition, DNA damage, and proteome instability. A number of studies suggest that the DNA repair process is less efficient with age, resulting in the development of genomic instability, which can lead to senescence [39,40]. Environmental stressors or injury can promote premature senescence, irrespective of normal aging [41]. Senescence significantly contributes to ECM alterations associated with physiological remodeling of the lungs in aging, via mTOR1-dependent mechanism(s) [42]. Studies from our lab have demonstrated that aged mice exhibit persistent senescence and non-resolving fibrosis in response to lung injury, suggesting that senescence is a pro-fibrotic mechanism in the context of aging [15]. Thus, cellular senescence may not only be a consequence of lung aging but also in itself contributes to accelerated lung aging. Numerous studies have demonstrated the accumulation of senescent cells in the pathologic tissue regions of human patients with age-associated fibrotic diseases, including IPF [15,[43], [44], [45], [46], [47], [48], [49]], liver fibrosis [50,51], kidney fibrosis [52], and cardiac fibrosis [53]. It should be noted that no single marker can be used to define a senescent cell [54], but rather a number of markers are associated with senescence (including senescence-associated β-galactosidase, p16, p53, p21, γH2A.X, and others), and typically a combination of these markers are used to identify senescent cells. The studies described above utilized various accepted methods to identify senescence in human patient samples. Increased telomere-associated foci (TAF), senescence-associated β-galactosidase, p16, p21, γH2A.X has been demonstrated in human IPF lung tissue [15,[43], [44], [45], [46], [47], [48], [49]]. Increased senescence-associated β-galactosidase and shorter telomere lengths were used to demonstrate senescence in hepatocytes from human cirrhotic liver tissue [50,51]. In human diabetic kidney tissue, increased senescence-associated β-galactosidase and p16 were utilized to identify senescent cells [52]. In human fibrotic cardiac tissue, increased senescence was demonstrated by senescence-associated β-galactosidase, p16, and p21 [53]. Overall, the increased prevalence of senescent cells at the sites of age-associated pathologies supports a role for senescence in the pathogenesis of these age-related fibrotic diseases, including IPF. These observations support a growing body of evidence suggesting that senescence drives age-associated pathologies in late life [55]. The fact that many different stimuli (which promote increased ROS) can lead to senescence suggests that senescence may be a common pathway in response to redox imbalance [56]. It is not known if ROS is the cause or consequence of aging, nonetheless, there is little doubt that oxidative stress plays a critical role in the pathogenesis of age-related lung diseases. The current challenge is to improve our understanding of how redox imbalance, aging, and disease are intertwined in order to identify therapeutic strategies that promote optimal ROS levels.

## 2 NADPH oxidases-structure, activation, and function

Nox/Duox enzymes are an evolutionarily conserved enzyme family that consists of seven members in mammals (Nox1–5 and Duox1–2) [[57], [58], [59], [60]]. The only known function of Nox is the generation of ROS, including O_2_^•−^ and H_2_O_2_. All Nox/Duox family members contain a homologous COOH-terminal flavoprotein domain consisting of an NADPH-binding region and a flavin adenine dinucleotide (FAD) binding region. Nox1-5 isoforms contain a hydrophobic NH_2_-terminal domain consisting of six transmembrane α-helices, where four heme-binding histidines are located in the third and fifth transmembrane domains [61]. Nox5 is unique in that within its NH_2_-terminal cytosolic domain; it contains four calcium-binding motifs. Duox1/2 contain a seventh transmembrane domain and two calcium-binding motifs within the cytosolic domain between the sixth and seventh transmembrane domains. Activation of Nox1-4 isoforms involves forming a heterodimer with the transmembrane protein p22^phox^ [62], while Nox5 and Duox activation requires specific phosphorylation and binding of calcium in their intracellular domains [63].

To date, Nox1 [11,[63], [64], [65]], Nox2 [11,[64], [65], [66], [67]], and Nox4 [17,68,69] have been implicated in the pathogenesis of IPF. In addition to Nox, another potential source of ROS implicated in fibrosis is mitochondria [70], although the relative contributions and cooperation between mitochondrial ROS and Nox localized in the mitochondria have not been elucidated [71]. Below, we have focused our discussion on the structural and functional characteristics of Nox1, Nox2, and Nox4.

### 2.1 Nox1

Nox1, the first identified homolog of Nox2, is most enriched in the colon [72]. Several previous studies suggest a role for Nox1-dependent ROS generation in the context of host innate defense mechanisms in the gastrointestinal tract [73,74]. Given its role in innate immune responses in the gastrointestinal tract, Nox1 may play a similar role in host innate immune response in the lung. However, the distribution and function of Nox1 in the lung remains largely unknown. Nox1 is expressed in a wide range of cell types in lung tissue such as pulmonary vascular smooth muscle cells (VSMCs), human pulmonary artery endothelial cells, pulmonary epithelial cells, and bronchial epithelial cells [72,[75], [76], [77]]. Nox1 expression is increased by various conditions (including pulmonary hypertension) and by stimuli (including nicotine) [72,[75], [76], [77]]. Nox1 interacts with p22^phox^ at the cell membrane and is constitutively activated by forming a complex with cytosolic factors including organizing subunit NoxO1 (homolog of p47phox), activating subunit NoxA1 (homolog of p67phox) [[78], [79], [80], [81]], and Rac1 [82].

### 2.2 Nox2

Nox2 (also known as Gp91 phox) was the first discovered Nox [83,84] and the most thoroughly studied Nox isoform. Nox2 is most abundantly expressed in phagocytes (neutrophils and macrophages), and, to a lesser extent, its expression is also reported in structural cells (mesenchymal cells, smooth muscle cells, endothelial cells, and airway epithelial cells) [85]. Nox2-dependent ROS plays well-established roles in facilitating the antimicrobial activity of phagocytes and mediating inflammatory responses. Nox2 constitutively forms a nonactive heterodimer with the transmembrane protein p22^phox^. Activation of the Nox2/p22^phox^ complex requires translocation of four cytosolic subunits such as p47^phox^ (organizer), p67^phox^, p40^phox^ and either Rac1 or Rac2 to the enzyme. Phosphorylation of p47^phox^ induces a change in its conformation that then enables binding between p47^phox^ and p22^phox^, thus promoting the interaction of other cytosolic factors with Nox2 [86]. The activation of Nox2 by regulatory subunits has been extensively studied and the subject of in-depth reviews elsewhere [77,87].

### 2.3 Nox4

Nox4 was originally discovered as NADPH oxidase homolog highly expressed in the human and mouse kidney [88,89]. Although Nox1-Nox3 represent highly evolutionally conserved subgroups of Nox, the 578 deduced amino acid sequence of Nox4 exhibits only 39% homology to Nox2 (gp91phox), with relatively highly conserved motifs in the membrane-spanning regions, and binding sites for heme, FAD, and NAD(P)H [88]. In the lung, Nox4 is ubiquitously expressed in various types of cells including macrophages [[90], [91], [92]] as well as structural cells (e.g., smooth muscle cells, endothelial cells, mesenchymal cells, and epithelial cells) [85]. Among other Nox isoforms, Nox4 is unique in its ability to predominantly release H_2_O_2_ [[93], [94], [95], [96], [97]] and its TGFβ-inducibility of H_2_O_2_ [17]. This feature is presumed to be attributed to a particular property of the N-terminal domain as the modulation of the extracytosolic E-loop can switch from H_2_O_2_ to O_2_^•−^ producing enzyme [98]. Nox4, in contrast to other Nox isoforms, is independent of cytosolic adaptor proteins and considered constitutively active, suggesting that Nox4-dependent ROS production is primarily controlled by the abundance of its expression level or by direct post-translational modification [93]. Nox4 forms a heterodimeric complex with the membrane protein p22^phox^ that enhances its ROS-generating activity [99]. Genetic ablation of p22^phox^ significantly diminishes its Nox4-dependent ROS generation [100]. Although p22^phox^ is a common binding partner for all Nox isoforms except Nox5, mutation analysis revealed a unique binding surface for Nox4 [96]. In vascular smooth muscle cells (VSMCs), Nox4/p22phox forms a multimeric complex with polymerase delta-interacting protein (Poldip2) that positively regulates Nox4-dependent ROS production and modulates Rho-dependent cytoskeletal reorganization and cellular migration [101]. Furthermore, Toll-like receptor 4 (TLR4), a known receptor for LPS, can directly interact with and mediate Nox4-dependent ROS generation and NF-kappaB activation when both Nox4 and TLR4 are overexpressed in HEK293T cells [102]. These studies highlight the distinct interaction of the key binding partners with Nox4.

## 3 Cell specific roles of Nox in fibrotic responses

Overproduction of ROS by Nox results in oxidative stress that damages tissues over time and may contribute to the development of chronic diseases late in life, including IPF. The consequences of oxidative stress within specific cell types occurs in a highly cell context-dependent fashion. A number of different cell types in conducting airways, pulmonary alveoli, and vascular wall have been shown to express specific subsets of Nox. Here, we focus our discussion on individual cell types known to generate Nox-dependent ROS, which have been implicated in the pathogenesis of IPF.

### 3.1 Lung myofibroblasts

Lung fibroblasts serve as stromal cells in the interstitial space between the epithelial and endothelial layers. In response to tissue injury, local fibroblasts proliferate and migrate to the site of damage, where they differentiate into myofibroblasts [103]. Myofibroblasts are responsible for the deposition of interstitial ECM components (e.g., fibronectin, type I and III collagen), a key characteristic of fibrosis [103]. The normal repair process is preceded by apoptosis of myofibroblasts and clearance of the ECM, which leads to the resolution of fibrotic scar tissue [104]. In contrast, the accumulation of myofibroblasts in the injured lung parenchyma (fibroblastic foci) is a key histopathological hallmark [105] and prognostic marker of IPF [106]. These myofibroblasts exhibit high levels of ECM deposition and contractile activity, which contribute to alveolar collapse, impaired lung compliance, and gas-exchange abnormalities, the major clinical characteristics of IPF. Therefore, promoting the clearance of myofibroblasts represents a promising strategy for developing therapeutic interventions for IPF.

**Fig. 1:**
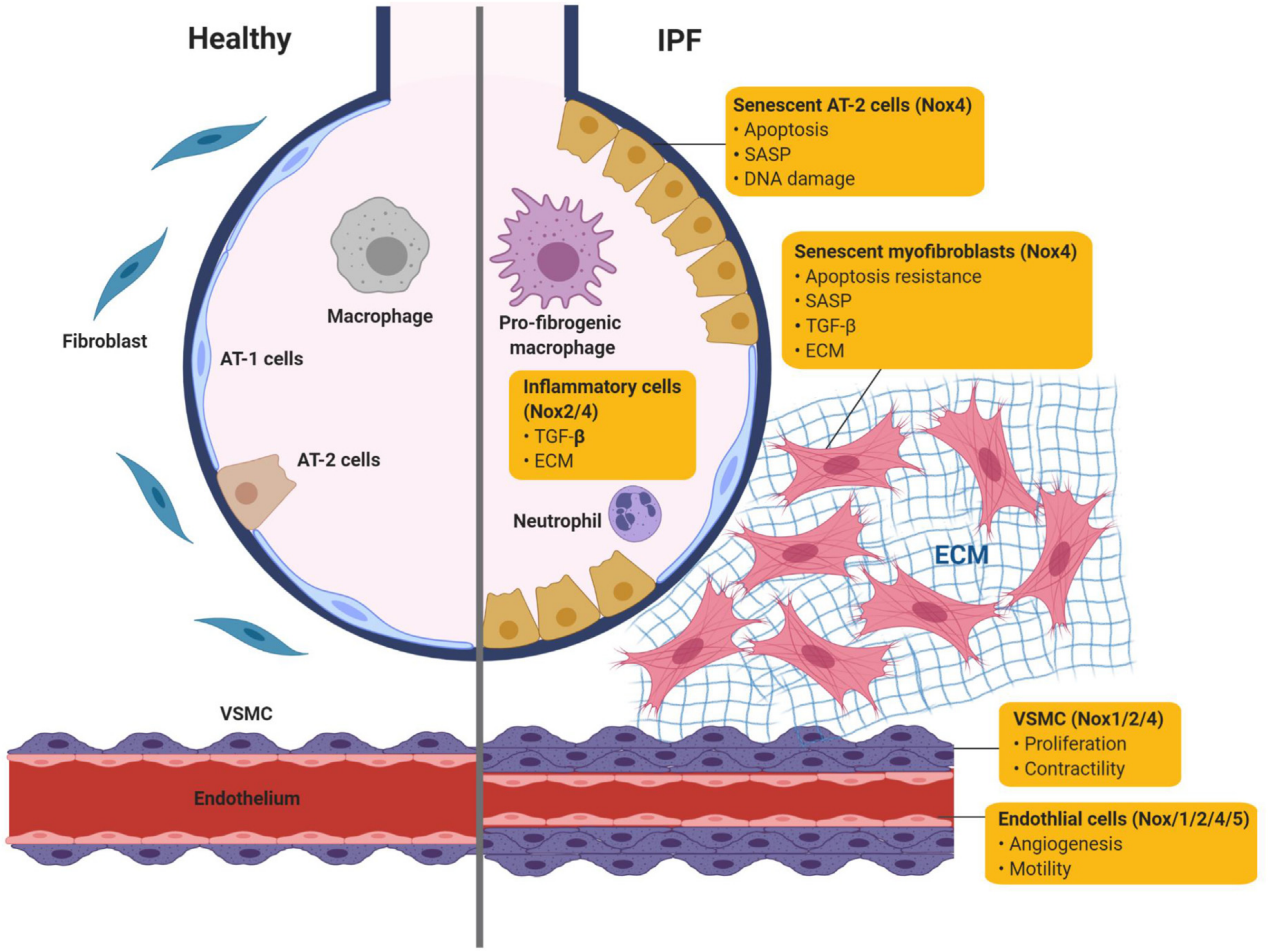
Potential source of ROS-generating Nox and function in the pathogenesis of IPF by each cell type. Nox isoforms are expressed in specific lung cells, where they mediate diverse functions in pathological repair processes in IPF lung. In senescent myofibroblasts, high levels of Nox4-dependent ROS generation can promote enhanced SASP, ECM deposition, contractility, and altered apoptosis susceptibility, which contributes to the accumulation of myofibroblast resulting in formation of fibroblastic foci in the interstitium of lung. In contrast, Nox4-dependent ROS generation can cause DNA damage and promote apoptosis in senescent AT-2 cells. Paracrine SAPS derived from senescent myofibroblasts and senescent AT-2 cells can spread cellular senescence and promote the profibrogenic responses. In lung macrophages, Nox4 mediates the profibrogenic polarization of lung macrophages that secrete TGF-β. Nox2-dependent ROS generation in neutrophils may contribute to the development of lung fibrosis while specific mechanism remains unknown. Nox4-dependent ROS generation may contribute to the development of pathological vascular remodeling by mediating proliferation/contractility of VSMC and angiogenesis/motility of vascular endothelial cells.

The most well-characterized and highly expressed Nox isoform in the lungs of patients with IPF is Nox4 [17,68,107]. Our group was the first to demonstrate a critical role for the Nox4 isoform in tissue repair functions of myofibroblasts and fibrogenesis (Fig. 1) [17]. TGF-β1, a fibrogenic cytokine known to be overexpressed in fibrotic disease [108], leads to the induction of Nox4-dependent ROS production, which promotes fibroblast migration [68] and mediates pro-fibrotic myofibroblast phenotypes, including differentiation, contraction, and ECM deposition [17]. Nox4-dependent ROS production by TGF-β1 is a highly specific and unique function of Nox4 [[93], [94], [95], [96], [97]]; no other Nox family gene members are affected at the transcript level [17]. In murine models of pulmonary fibrosis, suppression of Nox4 by siRNA or pharmacologic targeting of endogenous Nox by Diphenylene iodonium (DPI) (a well-validated pan-Nox inhibitor) or GKT137831 (putative dual Nox4/Nox1 small-molecule inhibitor) mitigate the development of fibrosis [17,107]. In addition, a recent study demonstrated that treatment with metformin, an anti-diabetic drug, attenuates TGF-β1–induced Nox4 expression, as well as subsequent ROS generation and myofibroblast differentiation in lung fibroblasts *in vitro* [109]. Further, intraperitoneal administration of metformin in mice was shown to attenuate bleomycin-induced lung fibrosis [109].

Myofibroblast senescence has been strongly implicated in IPF pathology and thus has been increasingly recognized as a therapeutic target for IPF [44,110,111]. Senescent myofibroblasts accumulate in fibrotic foci in the lungs of patients with IPF, and IPF-derived lung myofibroblasts demonstrate increased senescence *in vitro* [15,[43], [44], [45],^49^,^107^,[112], [113], [114]]. Myofibroblasts in injured tissues of young mice exhibit apoptosis susceptibility that permit fibrosis resolution, whereas myofibroblasts from aged mice acquire a senescent and apoptosis-resistant cellular phenotype that impairs the resolution of fibrosis [15]. Nox4 expression is elevated in senescent/IPF-derived lung fibroblasts [107], where Nox4-dependent ROS promotes cellular senescence and the acquisition of an apoptosis-resistant phenotype (Fig. 1) [15]. Genetic knockdown and pharmacological targeting of Nox4 in aged mice with established fibrosis promote a reversal of age-associated persistent fibrosis and improved survival [15]. Overall, these studies support the concept that age-associated redox-imbalance, mediated by dysregulation of Nox4, promotes pro-fibrotic myofibroblast phenotypes and persistent fibrosis in aging.

### 3.2 Alveolar epithelial cells

The alveolar epithelium consists of alveolar type-1 (AT-1) and alveolar type-2 (AT-2) epithelial cells that comprise ~8% and ~14% of all lung cells and cover about 96% and 4% of the internal surface area of the lung respectively [115]. AT-1 cells are thin (thickness 50–100 nm) squamous (diameter ~50–100 μm) cells that form an air-capillary barrier for gas exchange [116]. AT-2 cells are smaller (diameter ~10 μm) cuboidal cells with the characteristics of secretory cells, which produce surfactants. In the adult, AT-2 cells function as progenitor cells that can repopulate epithelium by self-renewal and differentiate into gas exchanging AT-1 cells following lung injury [115]. Impaired AT-2 cell integrity contributes to the pathogenesis of a variety of age-related lung diseases including chronic obstructive pulmonary disease (COPD), acute respiratory distress syndrome (ARDS), and IPF [[117], [118], [119], [120]].

The loss of alveolar epithelial cells appears to be a key characteristic of IPF lung tissue [[121], [122], [123]]. Repetitive epithelial cell injury has been implicated as a potential mechanism in the pathogenesis of IPF. Increased senescent of alveolar epithelial cells within fibroblastic foci has been reported in human IPF lungs, as well as in a number of animal models of lung fibrosis [15,43,[46], [47], [48],^124^]. Unlike senescent fibroblasts/myofibroblasts, senescent alveolar epithelial cells acquire a pro-apoptotic phenotype. TGF-β1 mediates primarily pro-apoptotic effects on alveolar epithelial cells, while it exerts the opposite effect on myofibroblasts [125]; this has been referred to as the “apoptosis paradox” [126]. The downstream signaling mechanisms of TGF-β1 that mediate differential apoptotic responses in these cells has yet to be fully elucidated. Alveolar epithelial cells that undergo trans-differentiation into mesenchymal cells (epithelial-mesenchymal transition or EMT) in response to TGF-β1 exposure appear to escape from this apoptotic fate; this could further impede re-epithelialization and amplify fibrotic response [127]. Another contrasting feature is the susceptibility of alveolar epithelial cells to ROS-induced injury [120], whereas myofibroblasts appear to be relatively resistant to similar levels of oxidative stress. In alveolar epithelial cells from IPF patients, oxidative stress contributes to DNA damage and increased apoptosis of [[128], [129], [130]]. H_2_O_2_ secreted from activated myofibroblasts can function as a diffusible death signal for the induction of lung epithelial apoptosis in IPF [130].

In the IPF lung, increased expression of Nox4 has been reported in atypical hyperplastic AT-2 cells (Fig. 1) [68]. Proof-of-concept for the contribution of Nox4-dependent ROS generation in abnormal alveolar epithelial cell repair in the context of the pathogenesis of IPF has been established in animal models [69,107]. In mice, the genetic ablation of Nox4 protected against bleomycin-induced pulmonary fibrosis with a concomitant reduction of alveolar epithelial cell apoptosis compared to WT type littermates [69]. Consistently, genetic ablation or pharmacological targeting of Nox4 with GKT136901 in murine primary alveolar epithelial cells significantly reduced TGF-β1-mediated ROS generation and mitigated apoptosis [69]. Together, these studies suggest that Nox4 may be expressed and activated in different types of alveolar space and contribute in different ways to fibrogenic responses.

### 3.3 Immune cells

Evidence from human and animal studies implicates the contribution of innate and adaptive immune cells in the pathogenesis of IPF. The complex role of innate immune cell populations and adaptive immune cells such as T-helper cells ad B cells in IPF is extensively discussed in a recent review [131]. Numerous studies demonstrate the presence of inflammatory cells infiltrate in IPF lung [[132], [133], [134], [135], [136]]. ROS released from inflammatory cells, primarily neutrophils and macrophages, may promote oxidant-mediated alveolar epithelial injury [136]. Nox2 is widely considered to have a limited, primarily phagocyte-specific tissue distribution such as neutrophils and monocytes/macrophages (Fig. 1) [137]. BALF neutrophils isolated from IPF patients exhibit elevated expression of p47phox and p67phox [138]. The genetic ablation of the p47^phox^ subunit of the Nox2 partially protected against the development of bleomycin-induced lung fibrosis in mice [139]. Overall, these studies suggest that Nox2-dependent ROS generation in phagocytes may play an important role in the pathogenesis of IPF. However, a recent study demonstrated that Nox4 mediates the profibrotic polarization of lung macrophages, where genetic ablation of Nox4 in macrophages led to decreased ECM deposition and protection from asbestos-induced pulmonary fibrosis [90]. Together, these studies suggest that Nox2-and Nox4-dependent ROS in innate immune cells may contribute to fibrogenic responses.

### 3.4 Vascular cells

In IPF patients, pulmonary hypertension (PH) is a common complication and strongly linked to the mortality [140]. PH affects approximately 10% of IPF patients in the early stage; however, as IPF advances, the incidence of PH increases up to 50% [140]. PH is characterized by extensive vascular remodeling, including altered proliferation, migration, differentiation, cytoskeletal rearrangements, and apoptosis of vascular cells such as endothelial cells and VSMCs. Abnormal vascular remodeling leads to narrowing and obliteration of the vessel lumen resulting in increased vascular tone. Under physiological conditions, ROS may have beneficial effects through the regulation of signal transduction and redox-sensitive gene expression. However, excessive ROS production is associated with the progression of vascular remodeling during pulmonary fibrosis [107,141,142]. The reduction of ROS by prostaglandin E5A (PDE5A) inhibitors can attenuate fibrosis-associated PH, both in human patients and a murine model of bleomycin-induced lung fibrosis [143,144]. Oxidative stress is likely to be involved in the pathophysiology of fibrosis-associated PH, however, the precise expression profile and contribution of Nox oxidases to vascular remodeling remain unknown.

There are three Nox oxidases found in VSMCs which include Nox1, Nox2, and Nox4 (Fig. 1) [145]. In IPF patients, upregulated Nox4 expression was found in thickened pulmonary arteries [146], suggesting a potential role for Nox4 in fibrosis-associated vascular remodeling. In support of this notion, in a rat model of bleomycin-induced fibrosis, treatment with GKT137831 resulted in protection from pulmonary vascular remodeling [107]. In VSMCs, Nox4 expression is increased in response to hypoxia via a hypoxia-inducible transcription factor (HIF-1α)-dependent mechanism [147,148]. Further, Nox4 plays a key role in the contractility and proliferation of VSMCs under hypoxic conditions [149,150]. These studies suggest that Nox4 may be dysregulated in VSMCs and potentially mediates vascular remodeling that leads to PH in the lungs of IPF patients.

The pulmonary endothelium serves as a tightly controlled barrier to prevent plasma exudation into the interstitium and alveolar space. The primary Nox isoforms expressed by vascular endothelial cells are Nox2 and Nox4 (Fig. 1) [151]. Nox4-dependent ROS regulates endothelial cell motility and angiogenesis, and genetic silencing of Nox4 attenuates hyperoxia-induced endothelial cell migration and capillary tube formation [152]. Hyperoxia has been shown to induce Nox1 [153] and Nox4 expression *via* Nrf2-dependent activation of the Nox4 promoter [154]. Nox2-mediated ROS in pulmonary artery endothelial cells has been implicated in the induction of autophagy, which contributes to impaired angiogenesis in persistent PH in fetal lambs [155]. Nox5 protein has been found in the endoplasmic reticulum of human microvascular endothelial cells [156], while its contribution to controlling the ROS-dependent process in the vascular remodeling remains elusive.

## 4 Emerging Nox-targeted therapeutics

Two drugs have gained FDA-approval for IPF, Pirfenidone (Genentech) and Nintedanib (Boehringer Ingelheim). Pirfenidone downregulates the production of growth factors and procollagens I and II [157]. Nintedanib is a tyrosine-kinase inhibitor, which targets proliferative pathways including vascular endothelial growth factor receptor (VEGFR), fibroblast growth factor receptor (FGFR), platelet-derived growth factor receptor (PDGFR), and type II TGF-β receptor [158]. However, these therapies only moderately slow the progression of lung decline, and there is no cure for IPF. Importantly, these therapies are associated with a number of significant and intolerable adverse effects. A study evaluating the combined results from two clinical trials with Pirfenidone reported that almost all patients in both studies (765 of 779; 98%) reported at least one treatment-emergent adverse event [159]. The most commonly reported adverse events from pirfenidone were gastrointestinal manifestations (nausea, dyspepsia, vomiting, and anorexia), skin disorders (rash, photosensitivity), and dizziness [159]. The most frequent adverse events of nintedanib treatment are gastrointestinal events, mostly diarrhea. A phase 3 clinical trial (including 663 patients) reported that 66.9% of patients treated with nintedanib reported diarrhea as a frequent adverse event, as compared to 23.9% in the placebo group. Further, a greater percentage of patients in the nintedanib group had adverse events leading to a permanent dose reduction (33.1%) and discontinuation (19.6%), as compared to the placebo group (4.2% and 10.3%, respectively) [160]. Furthermore, these therapies have a minimal impact on survival and do not improve the quality of life for IPF patients [[160], [161], [162]]. Clearly, improved therapies for the treatment of IPF and other fibrotic diseases are needed. It has been suggested that core pathways that mediate fibrosis in multiple organ systems may serve as better targets for anti-fibrotic drug development [163], and targeting age-associated redox imbalance has been implicated as one of these core pathways [164]. However, the ability to specifically and effectively target the source of oxidant generation remains challenging.

Since oxidative stress may involve excessive oxidant generation and/or impaired antioxidant capacities, therapeutic strategies have been directed inhibiting oxidant generation as well as stimulating antioxidant capacity. A number of antioxidant therapeutic strategies have shown promise in various preclinical models; however, these strategies have failed to demonstrate efficacy in the clinic [165]. For example, despite numerous promising pre-clinical studies with N-acetylcysteine (NAC), a well-studied antioxidant, a randomized clinical trial for IPF patients demonstrated no significant differences in decline of forced vital capacity (FVC), acute exacerbations, or mortality between NAC versus placebo [165]. There are a multitude of possible explanations for the relatively poor clinical translation antioxidant approaches. First, given the diverse roles of ROS, attempts to systemically scavenge ROS may hinder normal physiological signaling; this is evidenced by clinical studies where deleterious effects of untargeted antioxidant treatments have been reported [166,167]. Second, oxidants are highly reactive, and once generated damage may occur very quickly. Antioxidant strategies do not block the source of oxidative damage, which may, in part, explain the limited efficacy observed. Finally, it may be more effective to specifically target the primary enzymatic source of pathologically relevant ROS. Thus, inhibitors of specific Nox isoforms may prove to be more effective in comparison to antioxidant interventions for IPF. Although accumulating data support the concept that Nox play critical roles in fibrogenesis, the identification of *bona fide* Nox inhibitors remains challenging. Below, we have summarized major progress on putative Nox inhibitors.

DPI has been frequently used to inhibit ROS production mediated by various flavoenzymes, including Nox, cytochrome P450 reductase, eNOS, xanthine oxidase, and proteins of the mitochondrial electron transport chain [168]. To date, this nonspecific flavoenzyme inhibitor remains the most commonly used and well-studied Nox inhibitor. Despite its lack of specificity or other ROS-producing systems, DPI likely represents the most established and reliable example of a pan-Nox inhibitor. However, drawbacks that have prevented its therapeutic development include irreversible binding, lack of specificity, poor solubility, and toxicity *in vivo* [169]. Nonetheless, DPI remains a useful tool for Nox research.

VAS2870 was initially discovered by Vasopharm GmbH, as a Nox2 inhibitor [170]. Since then, VAS2870 has been commonly regarded as a pan-Nox inhibitor due to its ability to completely inhibit ROS production in multiple agonist-induced cell models with varied Nox expression [171,172]. Recent reports have shed light on the negative aspects of using VAS2870 as a Nox inhibitor, demonstrating off-target effects through thiol alkylation [173] and inhibition of mitochondrial respiration and cytotoxicity [174]. Thus, VAS2870 has an unspecific redox mode of action. Further, VAS2870 demonstrates cytotoxicity at low concentrations and it precipitates at higher concentrations [175].

Apocynin is a natural organic and nontoxic compound extracted from plants (*Apocynum*), which was characterized as an NADPH oxidase inhibitor in the early 1980s [176]. Apocynin is bioavailable *in vivo* [177] and shows therapeutic benefit in numerous mouse models of disease [178], and human asthmatic patients [179]. However, although apocynin is frequently used as a Nox2 inhibitor [[180], [181], [182]], numerous studies have demonstrated that apocynin is inactive for any Nox isoform but rather it shows intrinsic antioxidant activity, via ROS scavenging properties [174,175,183,184]. Thus, despite the potential therapeutic value of apocynin as an antioxidant, its beneficial effects cannot be attributed to Nox inhibition.

Ebselen, a seleno-organic compound, was initially identified as an antioxidant and glutathione peroxidase mimetic [185]. Since then, others have demonstrated that ebselen inhibits Nox2 and Nox5 [175,186]. However, ebselen acts as an antioxidant by reducing hydroperoxides [187] and/or direct scavenging of H_2_O_2_ [175] and inhibits other enzymatic systems [188,189]. It has a complex pharmacology, possibly due to the interaction and inactivation of protein cysteine residues. Further, ebselen is cytotoxic at low concentration, and its IC_50_ is close to its LD_50_ in living cells [175]. Because ebselen has a myriad of pharmacological activities, the inhibitory impact on Nox is challenging to define with certainty. Others have suggested that ebselen is not useful as a pharmacological agent to study Nox [175].

GKT137831 and GKT136901 were developed by Genkyotex (Geneva, Switzerland), which were identified by a high-throughput screening approach. GKT137831 is currently marketed as a dual Nox4/Nox1 small-molecule inhibitor drug candidate [190]. In Phase 2 studies of GKT137832 for diabetic nephropathy and primary biliary cirrhosis, the pre-specified primary endpoints were not met, although some of the secondary endpoints appeared promising [191] (www.clinicaltrials.gov; NCT02010242), although results have not been published. The drug appears to be well tolerated. In 2010, GKT137831 was granted orphan drug status for the treatment of IPF by the European Commission and is currently in clinical trials for IPF. A Phase 2 clinical trial of GKT137831 for IPF will be enrolling patients early this year, with the primary endpoint of reduction in circulating levels of an oxidative stress biomarker (NCT03865927) [192]. However, its specificity as a *bona fide* Nox4 inhibitor is controversial. A recent study performed a rigorous in-depth evaluation of these putative Nox inhibitors and demonstrated that Genkyotex drug candidates are, in fact, inactive as Nox inhibitors but rather they interfere with peroxidase-dependent assays [175]. In particular, these compounds non-specifically inhibit HRP, as they show similar inhibitory activity in the presence of H_2_O_2_ alone using a cell-free assay system [175]. Thus, the observed protective effects of Genkyotex compounds reported in pre-clinical *in vivo* studies [193] might not stem from the inhibition of Nox, but rather from an unspecific redox mechanism. It is also possible that these redox modifying compounds have downstream effects on Nox activity. Thus, although these compounds might be promising clinical candidates as redox modulators, their mechanism of action cannot be explained by direct Nox inhibition [175].

A number of groups (including our group) are currently working on the development of Nox inhibitors, however, *bona fide* Nox inhibitors have yet to reach the market. We refer to detailed reviews that describe in great detail about other less commonly used and/or unspecific inhibitors [175,[194], [195], [196], [197], [198]]. High-throughput screening approaches have frequently been used to identify Nox small-molecule inhibitors; however screening methods for Nox inhibitors typically utilize ROS detection-based screening assays that have limited specificity. Further, it is difficult to discern whether a putative inhibitor is acting directly on Nox vs. inhibition of a signaling pathway(s) leading to Nox induction/activation. A study reported that of >350 Nox inhibitors described, a majority of these did not directly block enzymatic activity, but rather they interfered with upstream signaling pathways or were ROS scavengers [199]. The examples provided above illustrate this point and underscore the complexity and challenges associated with identifying *bona fide* Nox inhibitors. Except for DPI, which lacks drug-like properties, reported Nox inhibitors mostly act as redox modifiers.

The ideal Nox inhibitor would need to fulfill several criteria: have no intrinsic antioxidant activity, does not inhibit other sources of ROS, should not interfere with upstream Nox signaling but rather inhibit Nox activity directly, and ideally would be selective for individual Nox isoforms. Any putative Nox inhibitor candidate would require rigorous false-positive testing to conclusively determine that it does not interfere with ROS assay mechanisms and/or exhibit cytotoxic effects (both of which can lead to false-positives) as well as independent validation. To date, Nox inhibitors lack specificity for a single isoform, and no selective Nox inhibitors have been identified. If/when Nox inhibitors become clinically available, it will become important to establish the long-term effects of Nox inhibition. It remains unclear whether long-term Nox inhibition for chronic illnesses, such as IPF, could be detrimental. However, Nox4 knockout mice are viable with no basal phenotype [69]. This is a reassuring observation from a drug targeting standpoint, as critical homeostatic roles for Nox4 in the unstressed state support lower risk for significant toxicity or side-effects from Nox4 inhibition. For now, the search for safe, selective, and effective Nox inhibitors continues.

## 5 Conclusions

IPF is an age-associated fibrotic lung disorder and a growing problem across developed countries with a rapid expansion of the elderly population. The disease is a fatal and progressive disorder with a median survival time of 2–4 years from diagnosis [200]. Although two drugs have gained FDA approval, the current drugs only moderately slow the progression of the disease. To date, no available therapies can “reverse” fibrosis and no drug treatment definitively improves the quality of life for patients. Numerous studies have implicated aging and oxidative stress in the pathogenesis; however, the age-associated pathologic mechanisms remain unexploited in drug development. Strategies aimed at specifically blocking the source of ROS through inhibition of NADPH oxidases, may prove to be more effective as anti-fibrotic therapies. Therapeutic strategies that target core pathways among age-related fibrotic diseases, including redox- and/or senescence-modulating agents, may represent promising clinical approaches that will improve survival and quality of life in patients with IPF.

## Funding

This work was supported by the Office of the Assistance Secretary of Defense for Health Affairs, through the Peer Reviewed Medical Research Program under Award No. W81XWH-17-1-0443 (LH), by the 10.13039/100000738Veterans Administration Health System grant 1 I01 BX003919-01A1 (LH), and the 10.13039/100000002National Institutes of Health grant 1R21AG054766-01 (LH)

## Declaration of competing interest

The authors declare that they have no known competing for financial interests or personal relationships that could have appeared to influence the work reported in this paper.
